# BUILDING AN EVALUATION FRAMEWORK FOR THE OREGON CENTER OF EXCELLENCE FOR BEHAVIORAL HEALTH AND AGING

**DOI:** 10.1093/geroni/igae098.3815

**Published:** 2024-12-31

**Authors:** Lindsey Smith, Julia Unsworth, Allyson Stodola, Dani Himes, Leah Brandis, Karen Cellarius, Paula Carder, Walter Dawson

**Affiliations:** Oregon Health & Science University, Portland, Oregon, United States; Oregon Health & Science University, Portland, Oregon, United States; Portland State University, Portland, Oregon, United States; Portland State University, Portland, Oregon, United States; OHSU, Portland, Oregon, United States; Portland State University, Portland, Oregon, United States; OHSU-PSU School of Public Health, Portland, Oregon, United States; OHSU, Portland, Oregon, United States

## Abstract

The Oregon Center of Excellence for Behavioral Health and Aging (OCEBHA) was established in 2024 with the mission to serve as a backbone organization, coordinating efforts to improve behavioral health services for older adults in Oregon. Housed at Portland State University and Oregon Health & Science University, the Center is focused on enhancing workforce training, supporting community partner organizations, advancing research, and promoting policies that improve access to care. We created an evaluation plan aligned with the Collective Impact model. The evaluation framework was developed to assess OCEBHA’s effectiveness in growing workforce capabilities, coordinating partnerships, promoting health equity, and driving systemic change. By constructing the Center’s role as a backbone organization, the evaluation plan was designed to measure both the process and impact of the Center’s efforts across multiple sectors. Key elements of the plan include the integration of community feedback, collaboration metrics, and health equity outcomes. This approach ensures that the evaluation reflects the complex, collaborative nature of the Center’s work and provides actionable insights for continuous improvement.

